# A Metamodeling Framework for Quantifying Health Damages of Power Grid Expansion Plans

**DOI:** 10.3390/ijerph16101857

**Published:** 2019-05-26

**Authors:** Mark D. Rodgers, David W. Coit, Frank A. Felder, Annmarie G. Carlton

**Affiliations:** 1 Department of Supply Chain Management, Rutgers Business School, Newark, NJ 07102, USA; 2 Department of Industrial & Systems Engineering, Rutgers University, Piscataway, NJ 07102, USA; coit@soe.rutgers.edu; 3 Department of Industrial Engineering, Tsinghua University, 30 Shuangqing Rd, Haidian District, Beijing 10084, China; 4 Center for Energy, Economic & Environmental Policy, Rutgers University, New Brunswick, NJ 07102, USA; ffelder@rutgers.edu; 5 Department of Chemistry, University of California-Irvine, Irvine, CA 92697, USA; agcarlto@uci.edu

**Keywords:** generation expansion planning, health damages, metamodeling, emissions, simulation, operations research

## Abstract

In this paper, we present an analytical framework to establish a closed-form relationship between electricity generation expansion planning decisions and the resulting negative health externalities. Typical electricity generation expansion planning models determine the optimal technology–capacity–investment strategy that minimizes total investment costs as well as fixed and variable operation and maintenance costs. However, the relationship between these long-term planning decisions and the associated health externalities is highly stochastic and nonlinear, and it is computationally expensive to evaluate. Thus, we developed a closed-form metamodel by executing computer-based experiments of a generation expansion planning model, and we analyzed the resulting model outputs in a United States Environmental Protection Agency (EPA) screening tool that approximates the associated human health externalities. Procedural guidance to verify the accuracy and to select key metamodel parameters to enhance its prediction capability is presented. Specifically, the metamodel presented in this paper can predict the resulting health damages of long-term power grid expansion decisions, thus, enabling researchers and policy makers to quickly assess the health implications of power grid expansion decisions with a high degree of certainty.

## 1. Introduction

Since 2001, electricity generation in the United States has followed an increasing trajectory as there has been increasing demand for uninterrupted access to electricity for various global industries that are vital to economic growth [1]. In order to satisfy this increasing demand reliably and economically, fossil fuels, such as coal and natural gas, are used as the primary sources of electricity, accounting for nearly two-thirds of the total energy supply [1]. However, since fossil fuels are burned to produce steam that is then used to power turbines that drive electrical generators, this produces air emissions such as CO_2_, SO_X_, NO_X_, volatile organic compounds (VOCs), and particulate matter [2]. As a result, ground-level ozone concentrations in the tropospheric region of the atmosphere increase as a result of photochemical reactions with emissions from fossil fuel plants. These by-products are health hazards, as they contribute to smog, which may ultimately lead to heart and chronic lung conditions like asthma, bronchitis, and emphysema [3]. Furthermore, air emissions from fossil fuels react with atmospheric water molecules to produce various forms of acidic precipitation and dry acidic deposition, known as acid rain. This occurs when sulfur dioxide and nitric oxides react with atmospheric water to generate sulfuric acid and nitric acid, which creates acid rain [4]. While acid rain does not pose any direct health hazards, fine acidic particulates in air contribute to heart and lung conditions, which result in health externalities or costs absorbed by consumers [4].

Based on the literature, one of the earliest approaches to quantifying health damages from the electricity sector was developed by Rowe et al. In this research, critical factors in computing externalities for electricity generation sources were assessed by utilizing the New York State Environmental Externalities Cost Study and computerized externality model (EXMOD). Using response surfaces in combination with EXMOD model outputs, the researchers were able to develop a model to predict health externalities from electricity generation by varying 15 different factors, including the selection of generation type, location, and operating characteristics [5]. Their research suggested that the most critical factors in developing a model framework were the selection and application of air dispersion models, selection of air pollution thresholds for health impacts, reduced life span risks associated with ozone exposure and long-term exposure to particulate matter, values for CO_2_ damages, and the value to be applied to increased risks of reduced life span for individuals age 65 or older. This research was extended by Thanh and Lefevre, where they applied an impact pathway approach (IPA) to relate the origin of environmental burdens to human health consequences. This relationship enabled the estimation of health damages of sulfur dioxide and particulate matter emissions from the electricity generation output of four power units using different fuels (lignite, oil, natural gas, and coal) at four locations in Thailand. Using response surfaces, their results suggested that these externalities were relatively small, but not negligible, as they ranged from 0.006 to 0.05 U.S. cents per kilowatt-hour (in 1995 dollars) [6].

Conversely, researchers have also studied the health benefits associated with emissions reductions from the electricity sector. For example, Burtaw et al. quantified the economic benefits of reduced air pollution in the U.S. related to greenhouse gas emissions mitigation policies in the electricity sector. In this work, researchers leveraged historical emissions data and a United States Environmental Protection Agency (EPA) screening tool to generate regression models of human health benefits. By conducting hourly, day-ahead electricity market simulations, and linking this output to the EPA screening tool, their research suggested that a tax of $25 per metric ton of carbon emissions would yield NO_X_-related health benefits of about $8 per metric ton of carbon reduced in the year 2010 (1997 dollars) [7].

While the electricity sector is the prime culprit of air emissions in the United States, researchers have also worked to study the broader impact of air quality on human health across multiple sectors including transportation and agriculture. As part of these efforts, Voorhees et al. conducted a sensitivity analysis using an industry standard air quality model, BenMAP, and analyzed the associated human health externalities. BenMAP, which stands for the Environmental Benefits Mapping and Analysis Program, is an EPA economic model used for estimating health effects and the associated externalities associated with changes in air quality. BenMAP achieves this by leveraging a geographic information system-based program to estimate population-level exposure rates and changes in incidences of health outcomes [8]. In the work done by Voorhees et al., they leveraged the BenMAP tool to model various climate change scenarios, and they quantitatively assessed the output [9]. Furthermore, the management consulting firm ICF International also used BenMAP as a resource to create a damage function, which was a response surface used to evaluate changes in health indices as a result of the emissions reduction goals proposed in the Clean Power Plan [10].

State-of-the-art literature related to this particular domain primarily focuses on quantifying the impact of power generation, primarily from fossil fuel sources, on air quality as well as the aggregate societal benefits of avoided emissions resulting from the integration of renewables into the generation portfolio. For instance, in China, Qin et al. found that replacing coal with synthetic natural gas coupled with carbon capture and storage technology in the residential sector would avoid approximately 32,000 pollution-related premature deaths by 2020 [11]. Huang et al. qualitatively studied the relationship between fuel consumption for electricity and the resulting contributions to ambient concentrations of major air pollutants from the power sector [12]. They extended their work quantitatively by studying five development scenarios in an air quality model [13]. Additionally, Peng et al. evaluated various decarbonization scenarios for the electric power grid in order to develop pathways to mitigate peaking emissions by 2030 in China while simultaneously maximizing air quality and health benefits [14]. More aligned to the research in this paper, Millstein et al. estimated the benefits associated with reduced air emissions resulting from increased wind and solar generation using various EPA screening tools [15]. Additionally, Kerl et al. introduced the air pollutant optimization model (APOM), which solved the hourly unit commitment model in the state of Georgia with the assistance of a reduced form air quality model [16]. To estimate their health impact costs, a baseline hourly unit commitment model, along with the inputs and outputs of two EPA models, were synchronized linearly to develop response surfaces, or regression curves, that predicted health impact costs as a function of decision variables in the unit commitment model. In the long-term planning space, Rodgers et al. studied the health benefits of adding emissions limits to multi-period, multi-region, generation expansion planning (GEP) problems using an EPA screening tool [3]. Furthermore, Rodgers et al. extended this research by applying a simulation-based optimization framework to solve the GEP problem that simultaneously minimized market costs and health damages from air emissions in the objective function. To include health damages in their model, a metamodel was developed to approximate health damages from NO_X_ and SO_2_ emissions associated with power grid capacity expansion decisions [17]. Specifically, health damages from simulated power grid capacity expansion plans were evaluated in the co-benefits risk assessment (COBRA) model. COBRA is an EPA screening model that helps state and local governments assess human health damages from air emissions at the county, state, regional, or national level [18]. After executing these computer-based experiments, a closed-form kriging metamodel that approximated health damages as a function of capacity expansion decisions was developed, which was used as a surrogate for health damages. While this research provides guidance on how to obtain an expansion plan under these circumstances, there is need for systematic framework to evaluate a metamodel and simultaneously select its parameters.

Drawing from the aforementioned state-of-the-art research, and because of the highly stochastic, nonlinear relationship between electricity generation and the resulting health damages, researchers often apply black box modeling techniques in these instances with little understanding of how decision variables impact the response in question [17]. This lack of system knowledge is intensified as the magnitude and scope of power systems planning decisions grows from hourly dispatch decisions to long-term capacity expansion decisions across multiple regions. In this paper, our primary objective is to close this gap by developing a step-by-step, analytical framework that accomplishes the following:

- Leverages computer-based simulations of a generation expansion planning model, where the model outputs are used as inputs into the COBRA tool to quantify the resulting health damages;
- Establishes a closed-form expression to predict the aforementioned health damages as a function of power grid expansion decisions using a kriging metamodel; and
- Provides a procedure to select metamodel parameters that maximize prediction accuracy.

## 2. Materials and Methods

To address the aforementioned challenges associated with approximating health damages from generation expansion planning decisions, we extended the work done by Rodgers et al. in [3,17]. Specifically, we proposed a detailed, systematic framework to establish a mathematical relationship that quantified health damages as a function of power grid expansion decisions while simultaneously considering prediction accuracy. Following the approach developed by Rodgers et al., we first formulated and solved a GEP model to determine the optimal technology capacity investment strategy that minimized market costs, including capital costs, fixed operation and maintenance costs, and variable costs of generation. Using this GEP model, we simulated key model parameters from a normal distribution, and we executed multiple computer-based experiments from the optimization model. We then evaluated the outputs of our simulations in the EPA’s COBRA tool.. Next, we applied a kriging interpolation model to our dataset to express the relationship between the covariates—electricity expansion decisions—and the response—human health damages. This kriging interpolation model, which is a geospatial estimation method that predicts the values of a random field at unobserved locations based on an interpolated function of observed samples, served as a metamodel or surrogate function that approximated the output of COBRA using input data generated from GEP simulations, thus, it simplified the ability to quantify human health damages resulting from power grid expansion plans. Since our metamodel was a function of computer-based experiments, there was a quantifiable amount of error in prediction. Lastly, to account for this, we proposed a cross-validation procedure to select the metamodel with the least prediction error.

**Figure 1:**
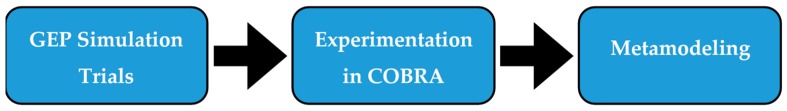
Metamodeling Procedure Flowchart. GEP: generation expansion planning; COBRA: co-benefits risk assessment.

In order to accomplish our research objectives, our first task was to establish a mathematical relationship to predict human health damages associated with electric power grid capacity expansion decisions, per the guidance of Rodgers et al., as given in Figure 1 [17].

To summarize, we generated expansion planning scenarios to be evaluated in an EPA screening tool, COBRA, to quantify human health externalities. A kriging metamodel was then applied to this data set, which predicted human health damages as a function of electricity dispatch decisions.

### 2.1. Simulation and Experimentation Procedure

To create a metamodel of human health damages in the context of GEP, we needed to generate a diverse sample space of data points from our expansion planning model to be evaluated in the COBRA tool. We referred to this process as seed generation.

**Figure 2:**
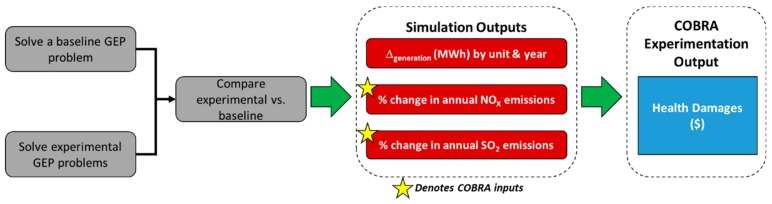
Simulation and Experimentation Procedure.

To initiate the seed generation process, as shown in Figure 2, first we solved a baseline GEP model. We then solved several experimental GEP models where key parameters were simulated from a normal distribution. For each experimental trial, we then compared the expansion decisions and obtained a change in MWh for each unit available within the system. Simultaneously, for each trial we obtained the percent change in NO_X_ and SO_2_ emissions from the baseline, which were used as inputs to be evaluated in the COBRA model.

#### Generation Expansion Planning (GEP) Model Formulation

We applied the GEP model used by Rodgers et al. to conduct our GEP simulation trials. The objective function of this GEP model was the sum of total market costs, which included investment costs, fixed operation and maintenance costs, and variable operation and maintenance costs, which were discounted over a predetermined time horizon [17]. The elements of the objective function are given below.

Investment Cost:

(1) $$O_{I}=\underset{y\in Y}{\sum }\frac{1}{{\left(1+r\right)}^{y}}\underset{r_{1}\in R}{\sum }\underset{i\in I}{\sum }\alpha_{yi}y_{yr_{1}i}$$

Investment costs are the capital costs associated with capacity expansion. $y_{yr_{1}i}$ is the capacity expansion decision, expressed as a continuous decision variable and given in MW, by generation unit type i in year y in region $r_{1}$. $\alpha_{yi}$ is the corresponding investment cost ($/MW). Additionally, we defined r as the interest or discount rate, *Y* as the set of all years in the planning horizon, *R* as the set of all regions in the network, and I as the set of all generating unit options.

Fixed operation and maintenance costs:

(2) $$O_{M}=\underset{y\in Y}{\sum }\frac{1}{{\left(1+r\right)}^{y}}\left[\underset{r_{1}\in R}{\sum }\underset{i\in I}{\sum }\left(\underset{u\left(\forall u\leq y\right)\in Y}{\sum }\beta_{ui}y_{ur_{1}i}\right)+\underset{r_{1}\in R}{\sum }\underset{i\in I^{*}}{\sum }\beta_{yi}q_{r_{1}i}\right]$$

Fixed operation and maintenance costs for unit i in year y are given by $\beta_{yi}$ in units of $/MW. In Equation (2), I* is the set of all existing generating unit options, I is the set of all available generating unit options, and $q_{r_{1}i}$ is the initial capacity of unit *i* in region *r*_1_. Additionally, we used the alias u as a surrogate notation for the year, y, to calculate the fixed operation and maintenance costs for all new capacity investments up to year y.

Variable operation and maintenance costs:

(3) $$O_{G}=\underset{y\in Y}{\sum }\frac{1}{{\left(1+r\right)}^{y}}\underset{t\in T}{\sum }\underset{r_{1}\in R}{\sum }\underset{i\in I}{\sum }v_{yti}x_{ytr_{1}i}$$

Variable operation and maintenance costs (including fuel costs) of generation from unit i during period t of year y are given by $v_{yti}$ in units of $/MWh. Each year is subdivided into six distinct time periods consisting of: (i) spring/fall-offpeak; (ii) spring/fall-peak; (iii) summer-offpeak; (iv) summer-peak; (v) winter-offpeak; and (vi) winter-peak, which span the set of all time intervals, *T*. $x_{ytr_{1}i}$ is the amount of aggregate electricity dispatched (MWh) from unit i in region $r_{1}$ in period t of year y.

Steam generation revenue:

(4) $$O_{R}=\underset{y\in Y}{\sum }\frac{1}{{\left(1+r\right)}^{y}}\underset{t\in T}{\sum }\underset{r_{1}\in R}{\sum }\underset{i\in I_{REV}}{\sum }\phi_{yi}x_{ytr_{1}i}$$

Annual steam generation (or cogeneration) revenue from combined heat and power (CHP) units is given by $\phi_{yi}$ in terms of $/MWh, and $x_{ytr_{1}i}$ is the amount of electricity dispatched from cogeneration units (in MWh) from in region $r_{1}$ period t of year y.

The total cost objective function, z, is given by Equation (5).

(5) $$z=O_{G}+O_{I}+O_{M}-O_{R}.$$

In our models, we minimized *z* across the network, subjected to system constraints. Definitions for GEP model sets, parameters, and decision variables are given in Table 1, Table 2 and Table 3.

The generation expansion planning formulation is given as follows:

$$\min\;z=O_{G}+O_{I}+O_{M}-O_{R},$$

and is subject to:

(6) $$\underset{r_{1}\in \Omega_{\left(y,r_{1},r_{2}\right)}}{\sum }l_{t}w_{ytr_{1}r_{2}}-\underset{r_{1}\in \Omega_{\left(y,r_{1},r_{2}\right)}}{\sum }w_{ytr_{2}r_{1}}+\underset{i\in I}{\sum }x_{ytr_{1}i}=d_{ytr_{1}}\;\;\;\;\forall y\in Y,t\in T,r_{2}\in R$$

(7) $$x_{ytr_{1}i}\leq \left(q_{r_{1}i}+\underset{u\left(\forall u\leq y\right)\in Y}{\sum }y_{ur_{1}i}\right)\delta_{t,i}h_{t}\;\;\;\;\;\forall y\in Y,t\in T,r_{1}\in R,i\notin I_{ND}$$

(8) $$x_{ytr_{1}i}\leq \left(q_{r_{1}i}+\underset{u\left(\forall u\leq y\right)\in Y}{\sum }y_{ur_{1}i}\right)\delta_{ti}h_{t}\eta_{tr_{1}i}\;\;\;\;\forall y\in Y,t\in T,r_{1}\in R,i\in I_{ND}$$

(9) $$w_{ytr_{1}r_{2}}-w_{ytr_{2}r_{1}}\leq \chi_{yr_{1}r_{2}}h_{t}\;\;\;\;\forall \left(y,r_{1},r_{2}\right)\in \Omega_{\left(y,r_{1},r_{2}\right)},Y,t\in T,i\in I$$

(10) $$w_{ytr_{2}r_{1}}-w_{ytr_{1}r_{2}}\leq \chi_{yr_{2}r_{1}}h_{t}\;\forall \left(y,r_{1},r_{2}\right)\in \Omega_{\left(y,r_{1},r_{2}\right)},Y,t\in T,i\in I$$

(11) $$\underset{i\notin I_{ND}}{\sum }q_{r_{1}i}+\underset{u\left(\forall u\leq y\right)\in Y}{\sum }\underset{i\notin I_{ND}}{\sum }y_{ur_{1}i}+\underset{i\in I_{ND}}{\sum }q_{r_{1}i}\theta_{i}+\underset{u\left(\forall u\leq y\right)\in Y}{\sum }\underset{i\in I_{ND}}{\sum }y_{ur_{1}i}\theta_{i}\geq d_{yi}^{*}m_{r_{1}}\;\;\;\forall y\in Y,r_{1}\in R$$

(12) $$\underset{r_{1}\in R}{\sum }y_{yr_{1}i}\leq \kappa_{y,i}\;\;\;\;\forall y\in Y,i\in I_{MAX}$$

(13) $$\underset{y\in Y}{\sum }\underset{r_{1}\in R}{\sum }y_{yr_{1}i}\leq \kappa_{i}^{*}\;\;\;\;i\in I_{MAX}$$

(14) $$x_{ytr_{1}i}\geq 0,w_{ytr_{1}r_{2}}\geq 0,y_{yr_{1}i}\geq 0\;\;\;\;\;\forall \left(y,r_{1},r_{2}\right)\in \Omega_{\left(y,r_{1},r_{2}\right)},Y,t\in T,i\in I$$

Equation (6) is the demand (or energy balance) constraint. For each of the periods in a given year, the total generation and transmission from both existing and new generating units should be at least as much as the corresponding demand in that region. Equations (7) and (8) are related to generating unit capacities for dispatchable and nondispatchable units. Equations (9) and (10) are the transmission network constraints. Equation (11) is the reserve margin constraint, and Equations (12) and (13) are investment limit constraints on an annual basis and throughout the time horizon of the model, respectively. Equation (14) specifies non-negativity for generation, transmission, and investment decision variables.

For each execution of the GEP model, the following key outputs were obtained and were used downstream as inputs into the COBRA tool:

- Generation (in MWh) in period *t* of year *y* of unit *i* in region *r*_1_;
- NO_X_ and SO_2_ emissions in region *r*_1_ in year *y*.

As previously stated, to create a metamodel of health damages as a function of power grid expansion decisions, a diverse sample space of data points (or outputs), via simulation, from our GEP model were evaluated in the COBRA tool. Following the procedure outlined in Figure 1, first we solved a baseline GEP model, which minimized total market costs. Concurrently, we solved additional experimental GEP models with the same model parameters as the baseline model but with simulated numerical values. Specifically, to obtain a diverse sample space, we varied several key parameters in the experimental model by simulating from a normal distribution with a coefficient of variation following a uniform random variable on the interval (0, 1). The parameters we varied in the GEP model were as follows:


Demand;Peak demand;Reserve margin;Minimum and maximum limits;Minimum renewable generation levels;Emissions rates

Emissions limits;Costs (investment, fixed, and variable);Steam revenue;Transmission capacity;Capacity factors for nondispatchable units; andDerating values.


For each GEP experimental trial, we computed the following values for every year in the planning horizon:

(15) $$\Delta x_{yr_{1}i}=\underset{t\in T}{\sum }\left(x_{ytr_{1}i,\;\;experimental}^{*}-x_{ytr_{1}i,\;\;baseline}^{*}\right)$$

(16) $$\Delta NO_{X}{}_{y,{\;r}_{1}}\left(\%\right)=\frac{NO_{X_{y,\;\;experimental}}-NO_{X_{y,\;\;baseline}}}{NO_{X_{y,\;\;baseline}}}$$

(17) $$\Delta SO_{2}{}_{y,{\;r}_{1}}\left(\%\right)=\frac{SO_{2_{y,\;\;experimental}}-SO_{2_{y,\;\;baseline}}}{SO_{2_{y,\;\;baseline}}}$$

Equation (15) defines the annual deviation in electricity dispatch of a given unit in a specified region of the experimental GEP model from the baseline model. Equations (16) and (17) quantify the annual percent deviation of NO_X_ and SO_2_ emissions, respectively, in a given region from the experimental trial to the baseline trial. We used the metrics from Equations (16) and (17) as inputs to the COBRA model, which allowed users to generate scenarios by specifying the changes in NO_X_ and SO_2_ emissions on an annual basis for the appropriate region. COBRA then estimated the corresponding changes in equivalent particulate matter (PM_2.5_) concentrations, using a source–receptor matrix, and calculated the estimated health damages based on embedded epidemiological and economic functions. Computer-based experiments in COBRA can be executed fairly quickly; however, the relationship between GEP decisions and the resulting health damages is highly stochastic and nonlinear, and thus is difficult to express in closed-form. As previously mentioned, black box methods are often utilized in these instances with little understanding of how decision variables impact the response in question. Using this computer-based experimentation approach, this resulted in a data set that was used for metamodeling, which established a closed-form relationship and closed this gap of information regarding how key variables impacted the response variable.

### 2.2. Kriging Metamodel Details

Kriging is an interpolation method that uses the observed data at all sample points to provide a statistical prediction of an unknown function by minimizing its mean squared error (MSE) [19]. To develop a kriging metamodel, the output of a deterministic computer experiment is treated as a realization from a stochastic process, which is then defined as the sum of a global trend function and a Gaussian stochastic process.

In a general sense, various metamodeling techniques have been used to solve complex optimization and decision-making problems. The simplest of these metamodel techniques leverages regression theory. For instance, by applying regression theory, experimental design, and feasible region partitioning approaches, Roux et al. investigated methods for obtaining more metamodels to approximate the value of an objective function [20]. Kalil et al. applied regression techniques along with factorial design in an industrial bioprocess optimization problem to maximize yield and productivity [21]. Quanhong and Calil applied regression methods to quantify the effect of liquid:solid ratio, NaCl concentration, and reaction time in order to maximize protein production from germinant pumpkin seeds [22]. They then used this regression model as a surrogate objective function in an optimization problem.

When the cost of evaluating the response of a system by experiments is computationally expensive, it is critical to use the most accurate metamodel for prediction purposes. In comparison to other metamodeling methods used in optimization, kriging provides the best prediction accuracy and serves as a more accurate and reliable surrogate objective function [23]. Furthermore, these models can be applied to surrogate systems to reduce the total cost of response evaluation [24]. Such metamodels have been deployed in black-box systems [24], metal-forming processes [25], aerodynamic design applications [26], and even multiobjective optimization models [27].

In our work, the kriging metamodel we utilized to quantify human health damages as a function of GEP expansion decisions was given by Equation (18) [17].

(18) $${\hat{\gamma }}_{yr_{1}i}\left(\Delta x_{yr_{1}i}\right)=\mu \left(\Delta x_{yr_{1}i}\right)+\overset{n\left(\Delta x_{yr_{1}i}\right)}{\underset{k=1}{\sum }}\omega_{k}\left[\gamma_{yi}\left(\Delta x_{yr_{1}ik}\right)-\mu \left(\Delta x_{yr_{1}ik}\right)\right]$$

${\hat{\gamma }}_{yr_{1}i}\left(\Delta x_{yr_{1}i}\right)$ is the predicted value of human health damages in year *y* for unit *i* in region *r*_1_, which is a function of a vector of unobserved dispatch values, $\Delta x_{yr_{1}i}$. $\Delta x_{yr_{1}ik}$ is a vector of observed dispatch deviations obtained from the experimental procedure, indexed by *k*. The observed value of $\Delta x_{yr_{1}ik}$ is given by ${\hat{\gamma }}_{yr_{1}i}\left(\Delta x_{yr_{1}i}\right)$, and $n\left(\Delta x_{yr_{1}i}\right)$ is the number of nearest neighbors to consider in the model. Kriging weight, $\omega_{k}$, is derived from the residuals, and the means of the predicted response and observed response are given by $\mu \left(\Delta x_{yr_{1}i}\right)$ and $\mu \left(\Delta x_{yr_{1}ik}\right)$, respectively.

Residuals, $R\left(\Delta x_{yr_{1}i}\right)$, in a kriging model have a stationary mean and covariance, where $R\left(\Delta x_{yr_{1}i}\right)=\gamma_{yi}\left(\Delta x_{yr_{1}ik}\right)-\mu \left(\Delta x_{yr_{1}i}\right)$, with $E\left[R\left(\Delta x_{yr_{1}i}\right)\right]=0$, and $Cov\left\{R\left(\Delta x_{yr_{1}i}\right),R\left(\Delta x_{yr_{1}i}+h\right)\right\}=C\left(h\right)$ for some lag *h*. In this case, C(h)=C(0)+SV(h), where we defined C(0) as the sill and SV(h) as the semivariogram. For this particular application, we employed the Gaussian semivariogram in Equation (19).

(19) $$SV\left(h\right)=\left(sill-nugget\right)\times \left(1-exp\left(\frac{-3h^{2}}{range^{2}}\right)\right)+nugget.$$

The Gaussian semivariogram function has two parameters that the user can specify—the sill and nugget. The sill is a stationary value of the semivariogram, where the slopes of the tangent lines become zero. The nugget is the value of the semivariogram function with zero lag. Additionally, the range is the lag value when the slope of the tangent lines becomes zero.

To select the parameters to be used in our metamodel, and to assess prediction accuracy, we applied a modified cross-validation procedure as follows:

- Step 1: We randomly partitioned the data set into eleven (11) equal subsets, designating one subset as a testing set and the remaining ten as validation sets to fit metamodels.
- Step 2: Using the validation sets identified in Step 1, kriging metamodels were fitted with a Gaussian variogram.
- Step 3: For each validation set, the mean absolute prediction error (MAPE) was computed against the test set.
- Step 4: All validation sets were aggregated into a single data set. This set was then used to build a full metamodel of the test set data and calculate the model error, as described in Step 3.

We repeated this procedure for using sill-to-nugget (STN) ratios ranging from 1 to 1.9 to fit our metamodels. We then plotted the MAPE as a function of the STN ratio, and we selected the STN value that minimized the MAPE for the full metamodel.

## 3. Results and Discussion

**Figure 3:**
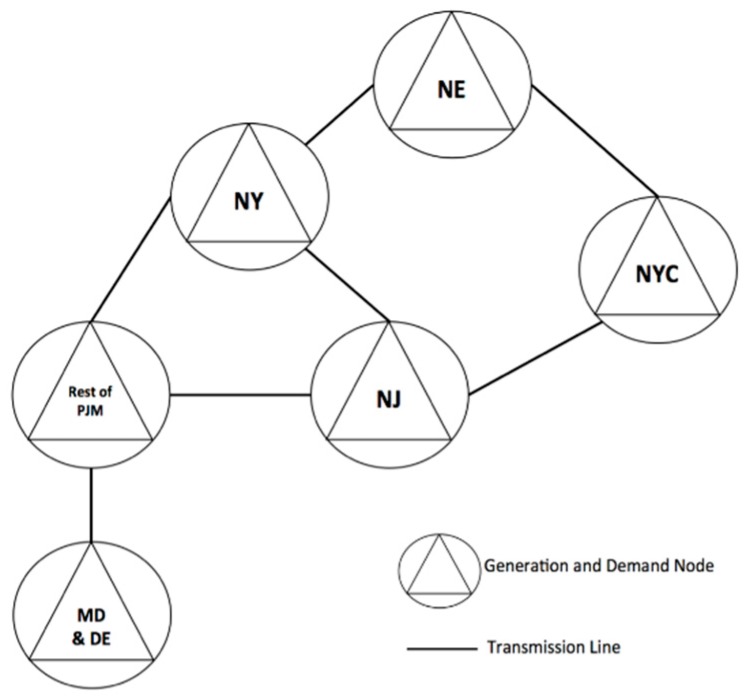
Transmission Network of the northeastern United States.

For the numerical example presented in this paper, we considered the northeastern United States, as shown in the representation in Figure 3 [3,17].

Our test network had six regions, given as follows:

- NE: New England (Maine, Vermont, New Hampshire, Massachusetts, Connecticut, and Rhode Island);
- NY: New York State (excluding New York City);
- NYC: New York City;
- NJ: New Jersey;
- MD & DE: Maryland, Delaware, and the District of Columbia;
- Rest of PJM: Illinois, Indiana, Kentucky, Michigan, North Carolina, Ohio, Tennessee, Virginia, and West Virginia.

Each region in the network had demand for electricity and also was capable of generating electricity. The available pool of generating unit technologies considered in our model comprised cycle gas turbines, coal, natural gas turbines, hydro, nuclear, petroleum, solar, biomass, on-shore wind, and off-shore wind. Additionally, connections between regions defined the transmission network. We considered a 25 y planning horizon (2015 through 2040). GEP model cost inputs are given in Table 4 (refer to Appendix A for a full list of GEP model assumptions and data sources).

Using the simulation procedure and the GEP model presented in Section 2, we executed ten simulation trials of our experimental GEP model, and we compared the results to a baseline GEP model to obtain changes in investment and dispatching decisions by unit and changes in NO_X_ and SO_2_ emissions. All GEP models were formulated as linear programs and solved using a CPLEX solver (an optimization package that leverages the simplex algorithm via the C programming language) in the generalized algebraic modeling system (GAMS) [30]. We then input these emissions changes into the COBRA model to quantify the health damages for each trial. Once we generated our data set, we then fit our kriging metamodel that predicted health damages. Our kriging metamodel was created using XonGrid, which is an open source interpolation tool [31].

Applying the cross-validation procedure presented in Section 2, we obtained the following prediction errors as a function of STN ratios displayed in Table 5.

**Figure 4:**
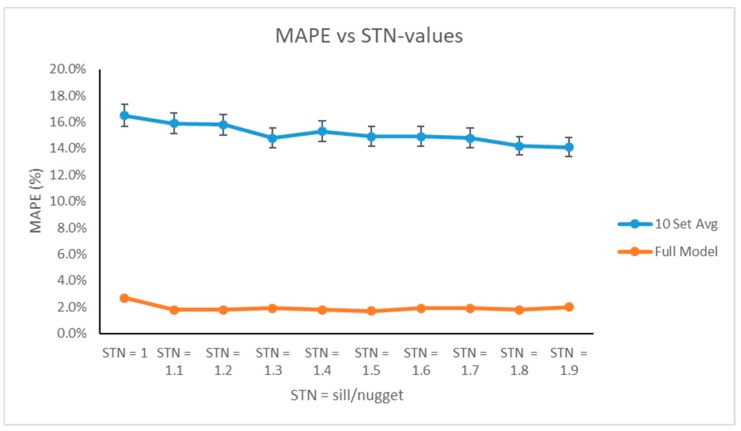
Mean Absolute Prediction Error Values from the Cross-Validation Procedure.

As evidenced by Table 5 and Figure 4, we executed the cross-validation procedure for various sill and nugget parameters in the Gaussian semivariogram. Based on the individual validation sets, on average, as we increased the STN-ratio, the prediction error decreased. Variability of the prediction error calculations was approximately 2%, which was relatively minor in the context of this problem. Upon applying the full data set to fit our metamodel, however, we noticed that the prediction error was significantly reduced and remained relatively stable for all values of *k*. Based on the full model, since the smallest error value occurred at STN = 1.5, this was the parameter we applied to the semivariogram and, thus, was used as an input parameter in our metamodel.

**Figure 5:**
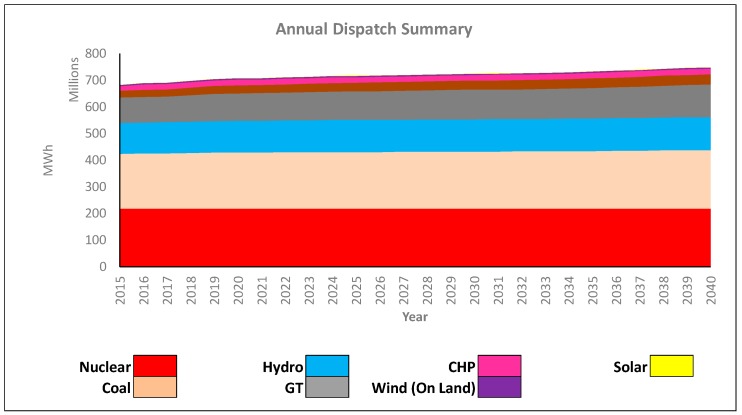
Baseline Model Dispatch Summary.

For comparative purposes, we displayed the results of the baseline model. Figure 5 displays the annual aggregate dispatch strategy associated with our baseline GEP model. In our network, base load units were nuclear, coal, hydro, and natural gas. Additional amounts of combined heat and power (or steam), solar, and wind (on land) were dispatched to satisfy the remainder of the demand. Table 6 gives the capacity investment strategy associated with our baseline GEP model, which suggested that we added over 17,000 MW of capacity to our network. The majority of these investments were in fossil fuel sources, as they were able to satisfy demand economically and reliably.

**Figure 6:**
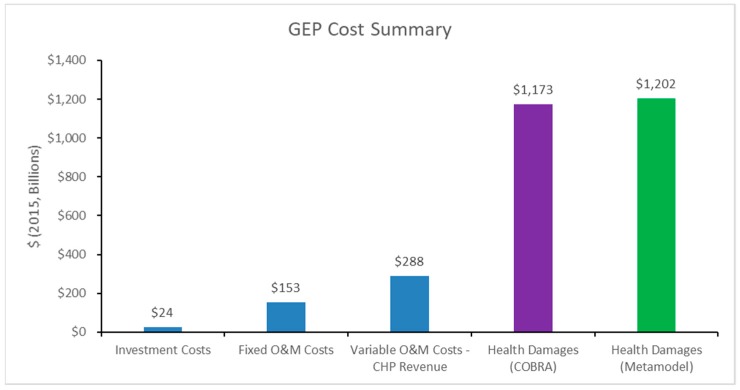
Total Cost Output Summary.

As given in the cost summary presented in Figure 6, market costs (specifically investment costs, fixed operation and maintenance costs, variable operation and maintenance costs, and combined heat and power (CHP) revenue) summed to roughly $465B, most of which were variable operation and maintenance costs. These costs were included in the objective function of our GEP and, thus, were optimized via the CPLEX tool. However, health damages of the resulting expansion plan were nearly three times the value of the optimized market costs. Additionally, the health damages predicted from our kriging metamodel were, globally, within 2.5% of the true values from COBRA.

**Figure 7:**
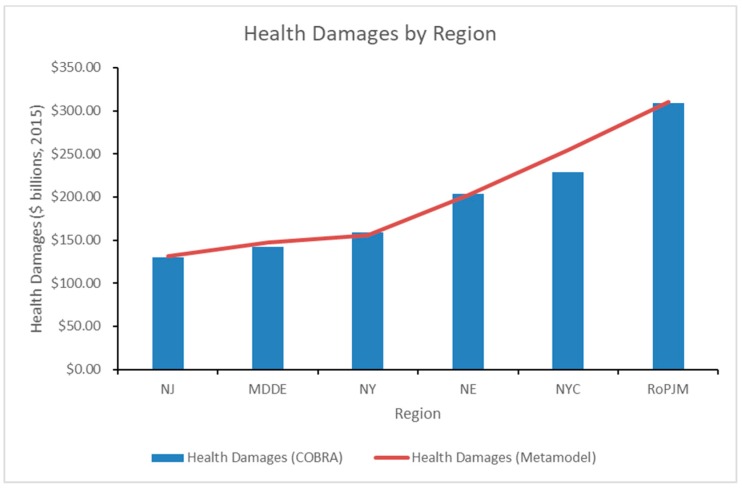
Health Damages by Region.

Figure 7 and Table 7 further detailed the breakdown of health damage values by region from the output of our baseline GEP model. Overall, there were areas where the model either over-predicted or under-predicted, depending on the region and generating unit. Regionally, the model over-predicted by approximately 11% in the NYC region and 4% in the MD & DE region, which was driven by the over-prediction of coal and nuclear damages, respectively. Additionally, the model slightly under-predicted health damages in the NY region by approximately 2%, which was driven by the under-prediction of natural gas damages.

## 4. Conclusions

This paper presents an extension of the research done by Rodgers et al. in [3,17], where health damages from generation expansion planning decisions are approximated. Specifically, an analytical framework to establish a mathematical relationship that quantifies health damages as a function of power grid expansion decisions, while simultaneously considering prediction accuracy, is presented. As demonstrated by the results, our metamodel successfully demonstrates the ability to approximate health damages as a function of GEP decisions with approximately 2.5% error, globally. This allows researchers and policy makers to quantify health damages as a function of power grid expansion decisions using a closed-form function, and it enables them to make more informed decisions on expanding power grid capacity.

Considering the output of the baseline model presented in Section 3, if not included in the objective function, health damages are nearly triple the optimized market costs. With the metamodeling framework presented in this paper, researchers can incorporate health damages in the objective function of a cost minimization GEP with the certainty of a high degree of prediction accuracy. Ultimately, this may yield significant investments in cleaner sources of energy, such as wind and solar technology, thus reducing health damages.

Another potential research extension would allow for a more detailed assessment of air quality and health damages by systematically linking more sophisticated air quality and economic models used by the EPA to our generation expansion planning model. One of the primary drawbacks of the reduced form air quality model COBRA is that it is based on simplified functions that translate air emissions, such as NO_X_ and SO_2_, into equivalent PM_2.5_ concentrations. Thus, in order to better estimate health damages, a more rigorous suite of screening tools can be used in place of the COBRA model to address this gap. Specifically, in place COBRA, we may substitute the EPA’s sparse matrix operator kernel emissions (SMOKE) model, which is a tool that allocates emissions both spatially and temporally. Using the SMOKE model output, we may use this data as inputs into the community multiscale air quality (CMAQ) model, which computes pollutant concentrations by using continuity equations [32]. Economic health implications of the associated pollutant concentrations can be assessed in the EPA’s BenMAP (Environmental Benefits Mapping and Analysis Program) tool, which approximates the health damages as a function of air quality effects [8]. Systematically linking these EPA models to our generation expansion planning model via the framework presented in this paper would not only yield more accurate emissions predictions, but it would also provide decision makers with a more detailed perspective of the health implications of expansion plans.

## Figures and Tables

**Table 1:** GEP Model Set Definitions.

*Y*	Years in the planning horizon (2015 through 2040)—indexed by y (or u as an alias)
*T*	Periods in the planning horizon (summer—peak/offpeak, winter—peak/offpeak, and spring/fall—peak/offpeak)—indexed by t
*I*	All generating units (nuclear, combined-cycle gas turbine, natural gas turbine, wind (land and offshore), biomass, coal, combined heat and power, solar, petroleum, and hydro)
I*	All existing units within the network—indexed by i
*I_MAX_*	Units with construction limits—indexed by i
*I_REV_*	Units with steam revenue (combined heat and power only)—indexed by i
*I_ND_*	Nondispatchable units (wind and solar)—indexed by i
*R*	Regions within the northeastern US network (New England, NY, NYC, NJ, MD & DE, and Rest of PJM)—indexed by $r_{1}$ (or $r_{2}$)
$\Omega_{\left(y,r_{1},r_{2}\right)}$	Set of transmission lines by year—indexed by ($y,r_{1},r_{2}$)
$\Phi_{\left(r_{1},r_{2}\right)}$	Network of regions for renewable trading—indexed by ($r_{1},r_{2}$)

**Table 2:** GEP Model Parameter Definitions

$\eta_{tr_{1}i}$	Capacity factor for nondispatchable units	$l_{t}$	Transmission losses by season
$\theta_{i}$	Capacity value by unit	$m_{r_{1}}$	Reserve margin by region
$\kappa_{y,i}$	Construction limits for each unit by year	*r*	Interest rate (3%)
$d_{ytr_{1}}$	Demand by period and region	$d_{yi}^{*}$	Peak demand by region and year
$\delta_{ti}$	Derating value by season and unit	$\phi_{yi}$	Steam revenue ($/MWh)
$\beta_{yi}$	Fixed cost ($/MW)	$\kappa_{i}^{*}$	Total construction limits by unit
$h_{t}$	Hours in each period	$\chi_{yr_{1}r_{2}}$	Transmission capacity by year
$q_{r_{1}i}$	Initial capacity by unit and region	$v_{yti}$	Variable costs ($/MWh)
$\alpha_{yi}$	Investment costs ($/MW)		

**Table 3:** GEP Model Decision Variables.

$x_{ytr_{1}i}$	Generation in period t of year y of unit i in region $r_{1}$ (MWh)
$y_{yr_{1}i}$	Capacity investment in year y in region $r_{1}$ of unit i (MW)
$w_{ytr_{1}r_{2}}$	Transmission of electricity in period t of year y from region $r_{1}$ to region $r_{2}$ (MWh)

**Table 4:** Generating Units and Their Costs, Capacities, and Emissions Rates.

Unit Type	^1^ Aggregate Capacity (MW)	^2^ Investment Costs ($/MW)	^2^ Maintenance Costs ($/MW)	^2^ Variable (Including Fuel) Costs ($/MWh)	^2^ Fuel Costs ($/MWh)	^2^ Steam Revenue ($/MWh)	^3^ NO_X_ (lbs/MWh)	^3^ SO_2_ (lbs/MWh)
Biomass	0	$4,359,382	$74,627	$30.29	$0.00	$0.00	4	10
Combined Cycle Gas Turbine (CCGT)	21,828	$1,096,358	$13,546	$69.37	$15.69	$0.00	2	0
Combined Heat & Power (CHP)	0	$1,744,772	$18,563	$75.86	$14.30	$56.63	2	0
Coal	59,968	$2,713,713	$38,327	$25.75	$12.91	$0.00	6	13
Natural Gas Turbine (GT)	16,172	$733,926	$12,198	$50.07	$15.69	$0.00	2	0
Hydro	24,082	$2,909,653	$12,198	$0.00	$0.00	$0.00	0	0
Nuclear	27,799	$4,326,537	$104,245	$6.35	$5.21	$0.00	0	0
Petroleum	4703	$1,114,480	$14,452	$99.31	$64.87	$0.00	4	12
Solar	446	$6,989,283	$13,523	$0.00	$0.00	$0.00	0	0
Off-Shore Wind	0	$4,459,051	$98,446	$0.00	$0.00	$0.00	0	0
Wind on Land	1985	$2,226,694	$35,088	$0.00	$0.00	$0.00	0	0

Notes: ^1^ [28]; ^2^ [1]; ^3^ [29].

**Table 5:** Mean Absolute Prediction Error (MAPE) Values.

	Set	STN Values = Sill/Nugget									
		1	1.1	1.2	1.3	1.4	1.5	1.6	1.7	1.8	1.9
MAPE Values	Set 1	15.8%	15.7%	14.8%	13.6%	14.5%	14.3%	14.6%	15.5%	12.2%	12.4%
	Set 2	17.1%	16.1%	14.3%	15.8%	12.9%	12.7%	12.8%	13.3%	14.0%	15.4%
	Set 3	13.7%	14.1%	15.9%	11.7%	12.0%	13.6%	14.0%	12.8%	12.8%	13.6%
	Set 4	14.1%	12.5%	14.0%	11.9%	11.4%	12.5%	12.2%	14.5%	12.7%	12.4%
	Set 5	19.1%	19.2%	21.0%	20.0%	22.6%	21.8%	20.8%	19.8%	17.4%	16.9%
	Set 6	18.1%	19.6%	19.3%	15.9%	16.8%	16.7%	15.2%	13.6%	15.2%	14.1%
	Set 7	20.0%	18.1%	16.7%	16.0%	18.9%	15.0%	15.8%	16.8%	15.5%	17.2%
	Set 8	14.6%	14.7%	12.7%	12.0%	12.9%	13.3%	13.3%	12.3%	12.2%	11.2%
	Set 9	17.0%	14.3%	15.2%	15.7%	16.9%	16.1%	15.2%	15.7%	16.3%	14.7%
	Set 10	15.8%	14.8%	14.2%	15.2%	13.6%	13.0%	14.9%	13.3%	14.2%	13.5%
	10 Set Avg	16.5%	15.9%	15.8%	14.8%	15.3%	14.9%	14.9%	14.8%	14.2%	14.1%
	Full Model	2.7%	1.8%	1.8%	1.9%	1.8%	1.7%	1.9%	1.9%	1.8%	2.0%

**Table 6:** Baseline Model Investment Strategy.

Region	Investment Summary by Region (MW)						
	GT	Coal	CHP	Petroleum	CCGT	Hydro	Totals
**MD & DE**	2348	404	648	709	717	738	5564
**NJ**	2379	1446	892	698	649	0	6065
**NYC**	2169	1420	1033	795	592	0	6009
**Total**	6897	3270	2574	2202	1957	738	17,637

**Table 7:** Comparison of Health Damage Values by Unit and Region.

Region	Nuclear			GT			Coal		
	Health Damages (COBRA) (in 2015 $, Billions)	Health Damages (Metamodel) (in 2015 $, Billions)	Error (%)	Health Damages (COBRA) (in 2015 $, Billions)	Health Damages (Metamodel) (in 2015 $, Billions)	Error (%)	Health Damages (COBRA) (in 2015 $, Billions)	Health Damages (Metamodel) (in 2015 $, Billions)	Error (%)
**NE**	$22.2	$23.1	3.9%	$18.5	$16.6	−11.0%	$163.3	$162.3	−0.6%
**NY**	$40.8	$36.2	−12.6%	$1.3	$1.1	−11.1%	$117.2	$118.2	0.9%
**NYC**	$0.0	$0.0	−	$25.0	$26.0	3.8%	$203.7	$228.6	10.9%
**NJ**	$32.0	$31.9	−0.4%	$45.8	$42.6	−7.4%	$52.6	$57.0	7.6%
**MD & DE**	$46.1	$49.2	6.2%	$6.6	$6.3	−5.7%	$89.0	$92.2	3.4%
**RoPJM**	$72.7	$73.3	0.8%	$30.5	$28.8	−5.7%	$206.0	$208.5	1.2%
**Totals**	$213.8	$213.7	−0.1%	$127.6	$121.4	−5.1%	$831.9	$866.7	4.0%

**Figure A1:**
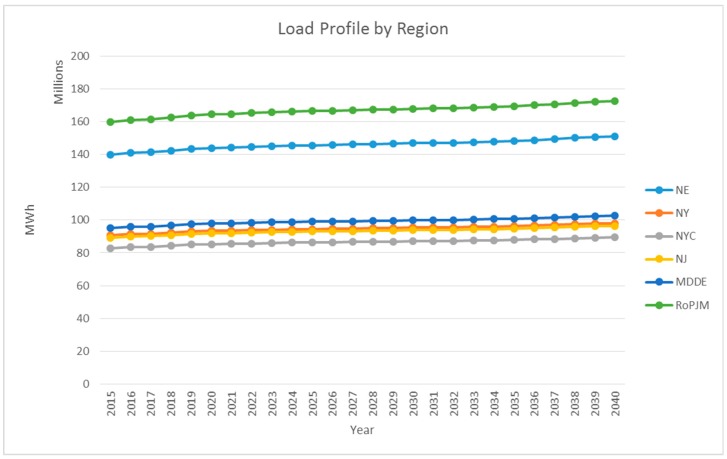
Load Profile by Region (U.S. Energy Information Administration, 2015).

## Appendix A. Additional Model Assumptions

**Table A1:** Initial Capacity by Region (in MW) (Cambridge Energy Solutions, 2011).

Region	Nuclear	Combined Cycle Gas Turbines	Coal (Low)	Coal (High)	Gas Turbines	Wind (On Land)	Petroleum	Hydro	Solar
**NE**	2873	203	2414	12,447	2110	0	3656	10,342	303
**NY**	5282	5313	4463	2763	305	1274	99	5705	-
**NYC**	-	2216	7765	-	5426	-	107	-	-
**NJ**	4119	6055	1241	2063	4816	8	47	406	13
**MD & DE**	5943	-	3419	1340	-	-	590	5348	128
**RoPJM**	9583	8041	4911	17,143	3515	704	204	2281	2
**Totals**	27,799	21,828	24,212	35,756	16,172	1985	4703	24,082	446

**Table A2:** Transmission Limits by Region (in MWh) (Cambridge Energy Solutions, 2011).

	Region	Receiving Region					
		NE	NY	NYC	NJ	MD & DE	RoPJM
**Transmitting Region**	**NE**	-	1420	-	-	-	-
	**NY**	-	-	-	950	-	-
	**NYC**	430	-	-	1685	-	-
	**NJ**	-	-	-	-	-	-
	**MD & DE**	-	-	-	-	-	5150
	**RoPJM**	-	2000		9268	-	-

**Table A3:** Unit Derating and Capacity Value Percentages (Cambridge Energy Solutions, 2011) (U.S. Energy Information Administration, 2015).

Unit Type	Derating Factor (%)	Capacity Value (%)
**Nuclear**	91%	100%
**Combined Cycle Gas Turbines**	84%	100%
**Gas Turbines**	86%	100%
**Wind (On Land)**	97%	32%
**Gas Turbines**	97%	100%
**Combined Heat & Power**	86%	100%
**Biomass**	89%	100%
**Coal (High)**	86%	100%
**Coal (Low)**	86%	100%
**Solar**	95%	14%
**Petroleum**	86%	100%
**Hydro**	95%	100%

**Table A4:** Unit Capacity Factors by Region (Cambridge Energy Solutions, 2011) (U.S. Energy Information Administration, 2015).

Region	Wind (On Land)	Wind (Off Shore)	Solar
**NE**	31%	59%	13%
**NY**	31%	57%	12%
**NYC**	31%	57%	14%
**NJ**	21%	39%	14%
**MD & DE**	25%	39%	14%
**RoPJM**	27%	43%	13%

**Table A5:** Reserve Margin Percentages.

Region	Reserve Margin Capacity (% of Peak Demand)
**NE**	116%
**NY**	117%
**NYC**	117%
**NJ**	115%
**MD & DE**	115%
**RoPJM**	115%

**Table A6:** Regional Greenhouse Gas Initiative (RGGI) CO_2_ Emissions Limits.

Year	Total Annual CO_2_ Emissions Limits in RGGI Regions (in lbs, Billions)
**2015**	366.6
**2016**	357.4
**2017**	348.5
**2018 through 2040 annually**	339.8

**Table A7:** Regional Emissions Limits (U.S. Environmental Protection Agency, 2013).

Region	NO_X_ Emissions Limits (in lbs, Millions)	SO_2_ Emissions Limits (in lbs, Millions)
**NE**	120.00	0.54
**NY**	120.00	0.26
**NYC**	120.00	0.26
**NJ**	120.00	0.13
**MD & DE**	120.00	0.36
**RoPJM**	120.00	1.07

**Table A8:** Annual Emissions Limits (U.S. Environmental Protection Agency, 2013).

Year	Total Annual SO_2_ Emissions Limits (in lbs, Billions)
**2015 through 2040 annually**	17.9

**Table A9:** Transmission Losses.

Period	Transmission Losses by Period (%)
**Summer-Peak**	91%
**Summer-Off-Peak**	91%
**Winter-Peak**	95%
**Winter-Off-Peak**	95%
**Spring/Fall-Peak**	93%
**Spring/Fall-Off-Peak**	93%

**Table A10:** Renewables Trading Network (General Electric International, 2014).

	Region	Receiving Region
**Transmitting Region**	NE	NE
	NY	NY
		NJ
		MD & DE
		RoPJM
	NYC	NYC
		NE
	NJ	NJ
		RoPJM
	MD & DE	MD & DE
		NJ
		NY
		RoPJM
	RoPJM	RoPJM

**Table A11:** Available Renewables by Region (General Electric International, 2014).

Region	Renewables Available by Region
MD & DE	Solar
	Biomass
	Wind (On Land)
	Wind (Off Shore)
NE	Biomass
	Wind (On Land)
	Wind (Off Shore)
	Solar
NJ	Biomass
	Wind (On Land)
	Wind (Off Shore)
	Solar
NY	Wind (On Land)
	Wind (Off Shore)
	Biomass
	Solar
NYC	Biomass
	Wind (Off Shore)
	Solar
RoPJM	Biomass
	Wind (On Land)
	Solar

**Table A12:** Minimum Percentage of Total Annual Dispatch from Renewables by Region (General Electric International, 2014).

Year	NE	NY	NYC	NJ	MD & DE	RoPJM
**2015**	11%	6%	6%	12%	12%	11%
**2016**	12%	6%	6%	13%	14%	14%
**2017**	13%	6%	6%	14%	15%	14%
**2018**	14%	6%	6%	16%	17%	15%
**2019**	15%	6%	6%	18%	18%	15%
**2020**	16%	6%	6%	20%	19%	16%
**2021**	16%	6%	6%	23%	19%	18%
**2022**	16%	6%	6%	23%	20%	18%
**2023**	16%	6%	6%	23%	20%	18%
**2024**	17%	6%	6%	23%	20%	18%
**2025**	17%	6%	6%	23%	20%	18%
**2026**	17%	6%	6%	23%	20%	18%
**2027**	17%	6%	6%	23%	20%	18%
**2028**	17%	6%	6%	23%	20%	18%
**2029**	17%	6%	6%	23%	20%	18%
**2030**	17%	6%	6%	23%	20%	18%
**2031**	17%	6%	6%	23%	20%	18%
**2032**	17%	6%	6%	23%	20%	18%
**2033**	17%	6%	6%	23%	20%	18%
**2034**	17%	6%	6%	23%	20%	18%
**2035**	17%	6%	6%	23%	20%	18%
**2036**	17%	6%	6%	23%	20%	18%
**2037**	17%	6%	6%	23%	20%	18%
**2038**	17%	6%	6%	23%	20%	18%
**2039**	17%	6%	6%	23%	20%	18%
**2040**	17%	6%	6%	23%	20%	18%

**Table A13:** Minimum Percentage of Total Regional Dispatch from Renewable Energy Sources (General Electric International, 2014).

Region	Biomass	Wind (On Land)	Solar
**MD & DE**	-	-	2%
**NE**	-	-	-
**NJ**	-	-	2%
**NY**	-	-	-
**NYC**	-	-	-
**RoPJM**	1%	8%	10%

**Table A14:** Annual Construction Limits by Unit (in MW per Region).

Year	Wind (On Land)	Combined Heat & Power	Nuclear	Wind (Off Shore)	Solar	Gas Turbines	Combined Cycle Gas Turbines	Petroleum
**2015**	10,000	10,000	0	0	10,000	10,000	10,000	10,000
**2016**	10,000	10,000	0	0	10,000	10,000	10,000	10,000
**2017**	10,000	10,000	0	0	10,000	10,000	10,000	10,000
**2018**	10,000	10,000	0	0	10,000	10,000	10,000	10,000
**2019**	10,000	10,000	0	0	10,000	10,000	10,000	10,000
**2020**	10,000	10,000	0	0	10,000	10,000	10,000	10,000
**2021**	10,000	10,000	0	10,000	10,000	10,000	10,000	10,000
**2022**	10,000	10,000	0	10,000	10,000	10,000	10,000	10,000
**2023**	10,000	10,000	0	10,000	10,000	10,000	10,000	10,000
**2024**	10,000	10,000	0	10,000	10,000	10,000	10,000	10,000
**2025**	10,000	10,000	0	10,000	10,000	10,000	10,000	10,000
**2026**	10,000	10,000	0	10,000	10,000	10,000	10,000	10,000
**2027**	10,000	10,000	10,000	10,000	10,000	10,000	10,000	10,000
**2028**	10,000	10,000	10,000	10,000	10,000	10,000	10,000	10,000
**2029**	10,000	10,000	10,000	10,000	10,000	10,000	10,000	10,000
**2030**	10,000	10,000	10,000	10,000	10,000	10,000	10,000	10,000
**2031**	10,000	10,000	10,000	10,000	10,000	10,000	10,000	10,000
**2032**	10,000	10,000	10,000	10,000	10,000	10,000	10,000	10,000
**2033**	10,000	10,000	10,000	10,000	10,000	10,000	10,000	10,000
**2034**	10,000	10,000	10,000	10,000	10,000	10,000	10,000	10,000
**2035**	10,000	10,000	10,000	10,000	10,000	10,000	10,000	10,000
**2036**	10,000	10,000	10,000	10,000	10,000	10,000	10,000	10,000
**2037**	10,000	10,000	10,000	10,000	10,000	10,000	10,000	10,000
**2038**	10,000	10,000	10,000	10,000	10,000	10,000	10,000	10,000
**2039**	10,000	10,000	10,000	10,000	10,000	10,000	10,000	10,000
**2040**	10,000	10,000	10,000	10,000	10,000	10,000	10,000	10,000

**Table A15:** Overall Construction Limits by Unit.

Unit Type	Maximum Construction Limit (MW)
**Wind (On Land)**	150,000
**Combined Heat & Power**	150,000
**Nuclear**	150,000
**Wind (Off Shore)**	150,000
**Solar**	150,000
**Petroleum**	150,000
